# Comparison of four functionalization methods of gold nanoparticles for enhancing the enzyme-linked immunosorbent assay (ELISA)

**DOI:** 10.3762/bjnano.8.27

**Published:** 2017-01-25

**Authors:** Paula Ciaurriz, Fátima Fernández, Edurne Tellechea, Jose F Moran, Aaron C Asensio

**Affiliations:** 1 Cemitec (Multidisciplinary Center of Technologies for Industry), Polígono Mocholí, Plaza Cein 3, Noain 31110, Spain; 2 IdAB-CSIC-UPNA-GN (Institute of Agro-Biotechnology), Public University of Navarre, Campus Arrosadía s/n, Pamplona 31006, Spain

**Keywords:** allergen, ELISA enhancement, functionalization, gliadin, gold nanoparticle

## Abstract

The enzyme-linked immunosorbent assay (ELISA) technique is based on the specific recognition ability of the molecular structure of an antigen (epitope) by an antibody and is likely the most important diagnostic technique used today in bioscience. With this methodology, it is possible to diagnose illness, allergies, alimentary fraud, and even to detect small molecules such as toxins, pesticides, heavy metals, etc. For this reason, any procedures that improve the detection limit, sensitivity or reduce the analysis time could have an important impact in several fields. In this respect, many methods have been developed for improving the technique, ranging from fluorescence substrates to methods for increasing the number of enzyme molecules involved in the detection such as the biotin–streptavidin method. In this context, nanotechnology has offered a significant number of proposed solutions, mainly based on the functionalization of nanoparticles from gold to carbon which could be used as antibody carriers as well as reporter enzymes like peroxidase. However, few works have focused on the study of best practices for nanoparticle functionalization for ELISA enhancement. In this work, we use 20 nm gold nanoparticles (AuNPs) as a vehicle for secondary antibodies and peroxidase (HRP). The design of experiments technique (DOE) and four different methods for biomolecule loading were compared using a rabbit IgG/goat anti-rabbit IgG ELISA model (adsorption, directional, covalent and a combination thereof). As a result, AuNP probes prepared by direct adsorption were the most effective method. AuNPs probes were then used to detect gliadin, one of the main components of wheat gluten, the protein composite that causes celiac disease. With this optimized approach, our data showed a sensitivity increase of at least five times and a lower detection limit with respect to a standard ELISA of at least three times. Additionally, the assay time was remarkably decreased.

## Introduction

Enzyme-linked immunosorbent assay (ELISA) is a technique based on the ability of antibodies to bind specifically to an antigen and has been used for more than 55 years [1]. Nowadays, it is the most commonly used technique for routine monitoring and analysis [2–3]. Initially, the antigen–antibody interaction was monitored by means of radioactive species, but soon these methods were replaced by easier to read and safer enzymatic systems, where is peroxidase (HRP) the most commonly used reporter enzyme due its stability and performance [2–4]. The success of ELISA relies on its detection limit, specificity, reproducibility and the possibility of high throughput screening, although the assay normally takes several hours to develop the response [3].

Despite all the advantages, the sensitivity of ELISA for certain systems is limited [5], pointing to the need for novel strategies that could improve the ELISA limit of detection (LOD). Some strategies have been explored to enhance sensitivity, such as redox complexes, electroactive molecules and metal ions [6]. Along these lines, several nanotechnology-based strategies have been proposed involving nanoparticle-based solutions [5,7–12]. Nanoparticles can serve as excellent carriers for specific recognition molecules such as antibodies or probes as well reporter molecules. Due to their high surface/volume ratio, they present more binding sites for capture elements and for reporting tags leading to amplification of the analytical signal in a single recognition reaction [6,8]. Luo and co-workers showed better sensitivities and shortened times for the detection of C-reactive proteins by using a quantum-dot-labeled immunoassay [13]. Accordingly, an improvement in sensitivity of 5,000 times for the detection of the ataxia telangiectasia mutated protein by functionalized multiwalled carbon nanotubes was observed by Zhang et al. [7].

However, the most significant improvements in signal have been rendered by gold nanoparticles (AuNPs), presenting promising unique chemical and physical properties, as well as biological compatibility [5,14–15]. AuNPs possess the advantages of easy synthesis and narrow size distribution together with an easy and efficient surface modification compatible with linkers or biomolecules [16].

A critical step for obtaining gold complexes is the conjugation of biomolecules to AuNPs. Increasingly, the process of loading biomolecules to the nanoparticle surface it is considered more important, as its properties or biochemical activity can be changed. It was shown that several parameters such as surface chemistry, pH, stabilizing agents as well as addition procedure strongly affect final coverage and efficiency of biomolecules [17–18]. Moreover, the AuNP–biomolecule binding can be completed by different procedures. Biomolecules can be simply adsorbed on the nanoparticle surface by means of electrostatic or hydrophobic interactions, leading to a high number of proteins per particle and random orientation of biomolecules [8,12]. Other studies reported more stable covalent immobilization, where a better control of particle coverage is achieved and even the binding orientation can be controlled [19–21]. Each of the described procedures present advantages and disadvantages such as leakage of non-covalently attached biomolecules or loss of biomolecule activity due to aggressive protocols [22–23]. Thus, an optimal conjugation strategy will depend on the final application. To the best of our knowledge, there are no specific studies on the effect of conjugation strategy on the potential of gold complexes to improve ELISA sensitivity. Hence, the main objective of this work is to compare, under similar conditions, different functionalization strategies in order to know which one is the best approach for this kind of application.

In this work, a simple model for detection of rabbit IgG by AuNPs conjugated to goat anti-rabbit IgG (Ab) and HRP (AuNPs-Ab-HRP) was assayed to elucidate the best conditions for biomolecule binding and ELISA enhancement. We explored the effect of four different described procedures for binding antibodies and HRP to AuNP surfaces in order to enhance ELISA sensitivity. Afterwards, the strategy which demonstrated better sensitivity was used for detection of gliadin from wheat gluten, one of the main proteins of wheat gluten [24]. Gluten refers to a group of proteins contained in wheat, barley and rye and is thought to be the cause of celiac disease (CD). CD is an autoimmune enteropathy that causes mucosal damage in the small intestine, leading to malabsorption upon intake of gluten containing food [25]. Consequently, it is essential to use a highly sensitive and specific technique for gluten analysis in food. Nowadays, the method internationally accepted by the Codex Alimenatarious Comission is the sandwich ELISA assay [24]. Therefore, any strategy that could improve the detection limit generates considerable interest.

## Results and Discussion

### Conjugation of anti-rabbit IgG and HRP to AuNPs by direct adsorption and directional conjugation

**Figure 1:**
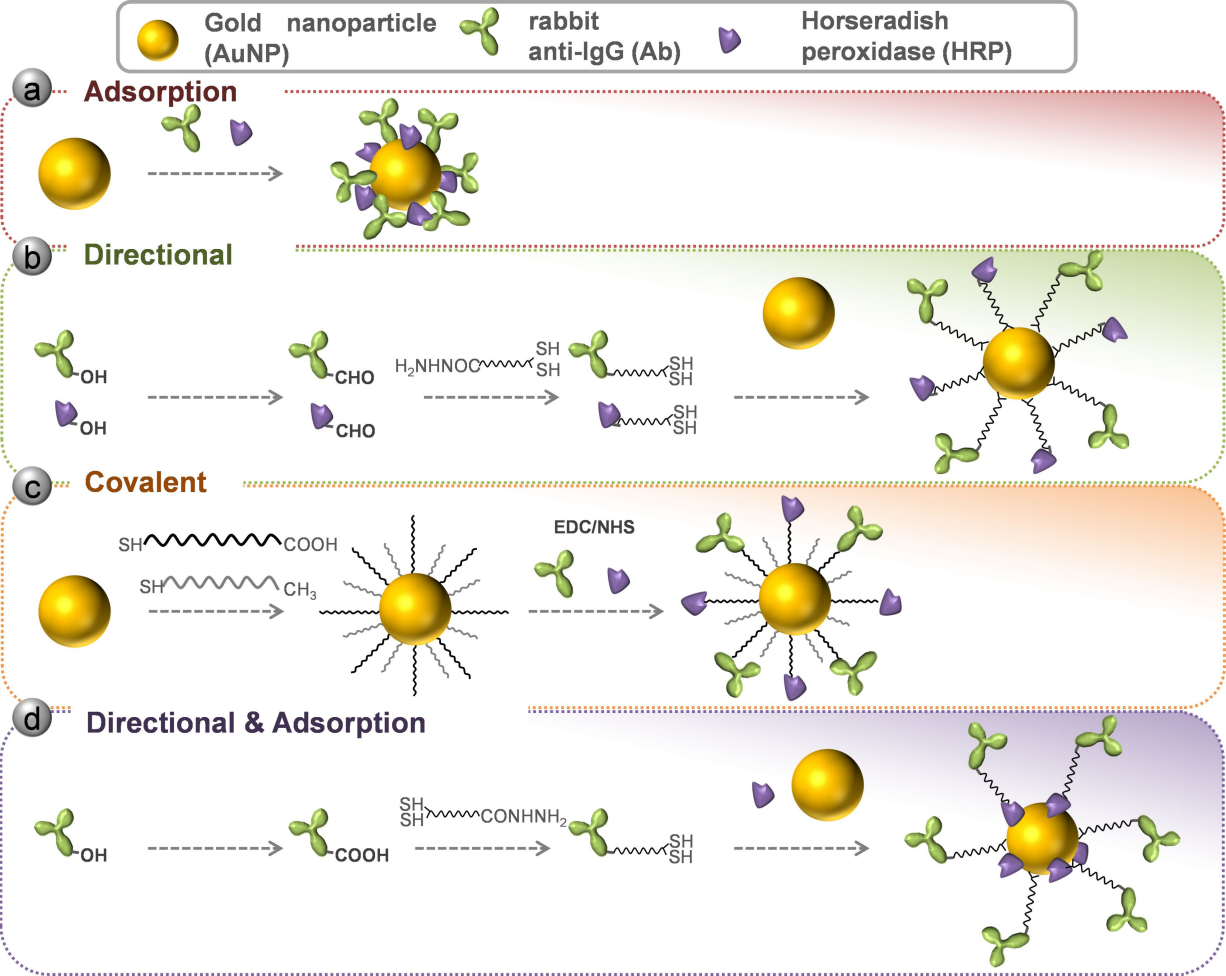
Schematic representation of the four different functionalization methods explored in this work. (a) Direct adsorption. (b) Directional conjugation with control of antibody and HRP orientation. (c) Covalent conjugation through antibody and HRP amine groups. (d) Combination of directional and adsorption strategy for antibody and HRP.

The aim of this work is to compare different AuNP functionalization methods in order to know which one is the best for enhancing the ELISA signal (Figure 1). As a first approach, two different strategies for conjugation of proteins to nanoparticles were evaluated: adsorption of biomolecules on nanoparticles by electrostatic/hydrophobic interactions or directional binding by means of a linker (Figure 1a,b).

For the first one (adsorption), a protocol was set up regarding previous work on the matter [8,26]. In the case of the directional strategy, a previously described protocol was followed [20]. A hetero-bifunctional linker, hydrazide-polyethylene glycol-dithiol, was used to control the orientation of the molecules on the surface of the nanoparticle. Hydrazide is able to react with aldehyde groups that can be generated by oxidizing the carbohydrates of glycosylated proteins, such as antibodies [20]. For this purpose, antibody and HRP carbohydrates were oxidized with periodate in order to attach the mentioned linker at the Fc region of the antibody. Modified HRP and antibodies were mixed with the AuNPs, triggering a covalent binding.

### HRP/Ab ratio optimization for direct adsorption and directional conjugation

**Figure 2:**
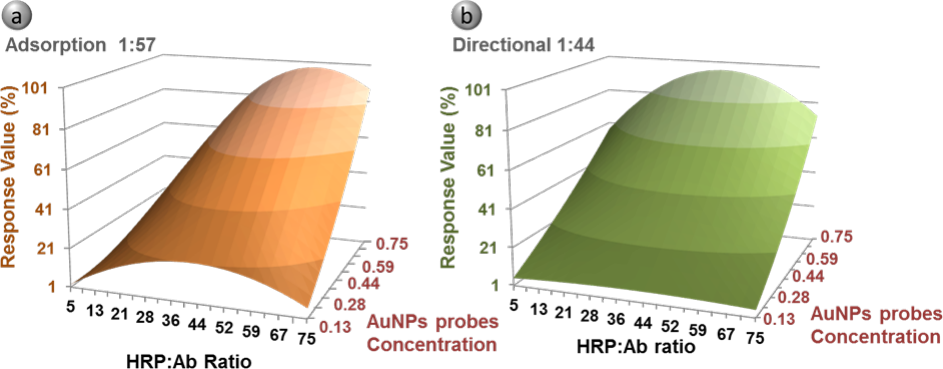
DOE experimental results for adsorption (a) and directional (b) methods. Estimation of the effect on response of HRP/Ab ratio and AuNP probe concentration. The layout displays the response value as percent of the maximal HRP signal at 450 nm. Coefficient of determination of DOE results *R*^2^ = 0.9485 (a) and *R*^2^ = 0.9768 (b).

In order to elucidate the best conditions for nanoparticle and biomolecule assembly, the HRP/Ab molar ratio is known to be one of the most influential parameters in AuNP complexes as well as the probe concentration [6,8,12]. Furthermore, to cover all the possible combinations of parameters, while keeping the number of calculations to a minimum, a design of experiments technique (DOE) [27] was applied. Through DOE, the influence of the HRP/Ab ratio and AuNP concentration on ELISA performance can be easily studied. The DOE experiment was performed using different ratios between HRP and goat anti-rabbit IgG (1:5, 1:40 and 1:75 HRP/Ab) to elucidate the best conditions for the two functionalization strategies evaluated, that is, direct adsorption of biomolecules and directional assembly. These conjugates were evaluated with a fixed concentration of rabbit IgG (1 µg/mL) coated in a microplate well. Moreover, the influence of AuNP probes at different dilutions was also considered. For each HRP/Ab ratio, three different concentrations of AuNP probes (0.05, 0.4 and 0.75 AU) were assayed. The results were evaluated in terms of percent with respect to maximal signal at 450 nm. As a result, optimized ratios of 1:57 and 1:44 were obtained for the adsorption and directional methods, respectively (Figure 2).

These ratios are close to similar works performed with carbon nanotubes and covalent conjugation of HRP and anti-IgG, where an optimal ratio of 1:50 HRP/Ab was also found [7]. However, they differ from the work of Zhou and coworkers, which was developed using 20 nm AuNPs and a direct adsorption strategy, where they found 1:6 as the optimal ratio for HRP/Ab [8]. A similar ratio (1:3) was selected by Wu et al. when modifying 15 nm AuNPs for the detection for *Samonella typhimurium* [5]*.* These differences could be due to longer incubation times in the mentioned references, around 3 and 24 hours respectively, compared to 1 hour applied in our protocols. In addition, the ratios HRP/Ab assessed by these authors are lower than the ones considered in our work. Besides the dissimilar procedures employed, it is described that different variations in ionic strength, pH, protein order addition, as well as the inherent protein properties may modify the amount of biomolecules bound to the nanoparticle surface [17,21,28]. In this study, where two different biomolecules meet at the AuNP surface, the surface chemistry, different affinities towards gold and the microenvironment may have a great influence on the antibody nature and affinity for the antigen. This underlines the need of a simultaneous comparison between different strategies in order to obtain the most suitable protocol for this particular application.

On the other hand, as mentioned, the concentration of gold complexes must be taken into account for enhancing the ELISA signal. Therefore, in DOE experiments the influence of increasing the AuNP concentration (0.05, 0.4 and 0.75 AU) was also assessed. As seen in Figure 2, the increase of AuNPs results in a better performance up to the concentration assayed. Thus, as a first approach, a concentration of 0.5 a.u. AuNPs was applied in the ELISA characterization. Nevertheless, the influence of complex dilution was further assayed with the selected functionalization strategy.

### Adsorption and directional strategies: comparison by ELISA

**Figure 3:**
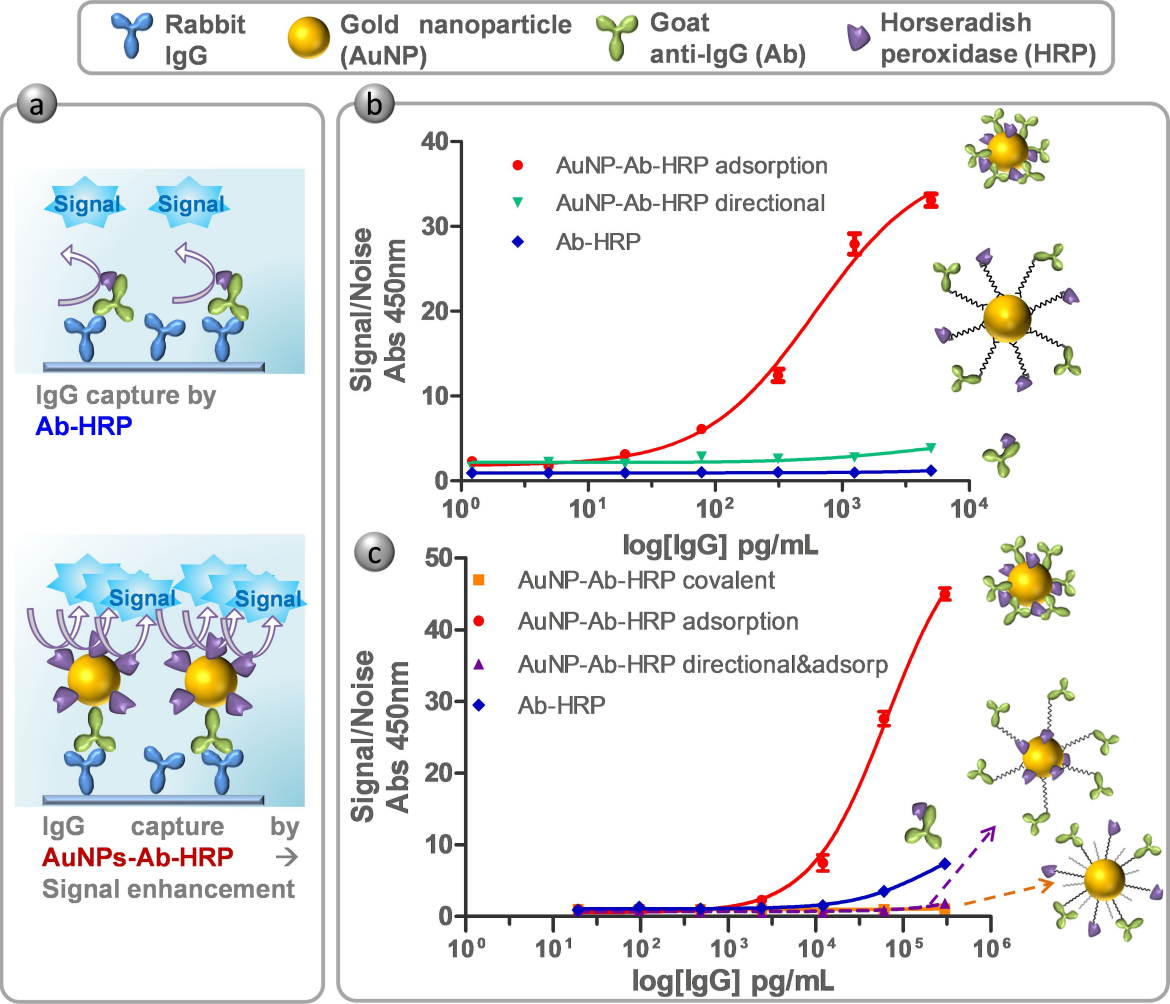
(a) Schematic representation of model ELISA and the basis of enhancement by means of AuNP probes. (b) Result of ELISA comparing AuNP probes prepared by adsorption (red curve), directional conjugation (green) and labelled secondary antibody (Ab-HRP, blue). (c) Result of ELISA comparing AuNP probes prepared by adsorption (red), covalent (orange), directional and adsorption (purple) and Ab-HRP (blue). Results are represented as the S/N ratio which represents the absorbance at 450 nm of samples in the presence and absence of IgG, respectively.

Conjugates were assayed by ELISA using rabbit IgG as the target. In all cases, AuNP probes were compared to a regular anti-rabbit IgG HRP conjugated antibody (Ab-HRP) to compare the sensitivity reached with the different methodologies (Figure 3a,b). Accordingly, the results were evaluated in terms of signal/noise (S/N) which represents the absorbance at 450 nm of samples in the presence and absence of IgG, respectively. The S/N ratio of samples conjugated by adsorption showed a higher response than directional conjugates or Ab-HRP. This was an unforeseen result, as better efficiency was expected due the directional conjugation, where more antigen-binding sites on the fragment antigen-binding (Fab) portion of the antibody are directed outward from the gold surface and therefore available for antigen binding [19–21]. Periodate is widely used for HRP conjugation to biomolecules [2,29–30]. For this reason, we considered it appropriate to follow the protocol of Kumar et al. [20] for directional functionalization of AuNP with Ab and HRP. However, this kind of protocol may cause partial enzyme denaturation, as periodate is a powerful oxidant and could decrease HRP activity to a great degree [2].

### New biofunctionalization strategies with covalent and directional/adsorption: comparison by ELISA

As described above, we decided to evaluate two other new approaches (Figure 1c,d). One approach is covalent conjugation, where antibodies and HRP are covalently bound to the surface by the means of a PEG linker through its free amine groups using the EDC/NHS carbodiimide method [30]. The second is a merge of the previously assayed procedures, combining the directional binding of the antibody with the adsorption of the HRP to the AuNP surface (directional and adsorption procedure). For the covalent strategy we set up the conjugation procedure according to previous works [19] and the DOE results. In the case of the directional/adsorption procedure, the protocol for directional Ab loading and concentration of HRP from adsorption method were applied.

Consequently, ELISA was assessed for comparing the new proposed strategies as well as the direct adsorption that already yielded good results (Figure 3c). Once more, adsorption conjugation resulted in better S/N response than the Ab. Surprisingly, new conjugation strategies (covalent and directional/adsorption) resulted in worse S/N values than direct adsorption and even more than Ab-HRP, although it is described that covalent and site specific immobilization leads to more stable and better defined composition conjugates [19–20,31]. In an attempt to better understand these data, it was found that the HRP molecule (Uniprot accession number P80679) presented a lower number of free amine groups (few lysine amino acid residues) compared to the antibody molecule. The lower availability of free amino groups could hamper the attachment of peroxidase in the covalent strategy (Figure 1c), although more experiments should be performed to confirm this. Consequently, this would lead to lower peroxidase coverage and thus lower ELISA enhancement. The combination of the directional and adsorption strategy would be presented as best alternative according to this hypothesis, however, ELISA experiments showed a low S/N ratio compared to other methodologies. The combined strategy implies a two-step functionalization, where the antibody is first directionally bound to the surface, and secondly, HRP is added for a direct adsorption loading. The sequential procedure inevitably signifies less free binding sites on the nanoparticle surface after the first step. It was previously described how the arrangements of biomolecules can affect complex coverage and behavior [17]. Moreover, Marie-Eve Aubin-Tam and coworkers showed how ligand charges around the particle can strongly influence protein structure, and therefore, activity [32]. Both factors would indicate lower peroxidase coverage/activity in this functionalization strategy.

In contrast, direct adsorption often leads to protein multilayers, as biomolecules have numerous residues which can non-specifically adsorb on AuNP surfaces [33]. Gagner et al. described how high protein loading resulted in lower loss of protein activity and secondary structure [34]. They assumed that subsequent adsorption of protein in multilayers allowed the conjugate to recover activity and remain stable. Taking into account the published results and considering our data, we postulated that the total number of proteins bound to AuNPs could probably be higher by the direct adsorption method than for the others strategies, resulting in lower protein denaturation and a higher S/N ratio.

### Optimization of AuNP concentration in ELISA

**Figure 4:**
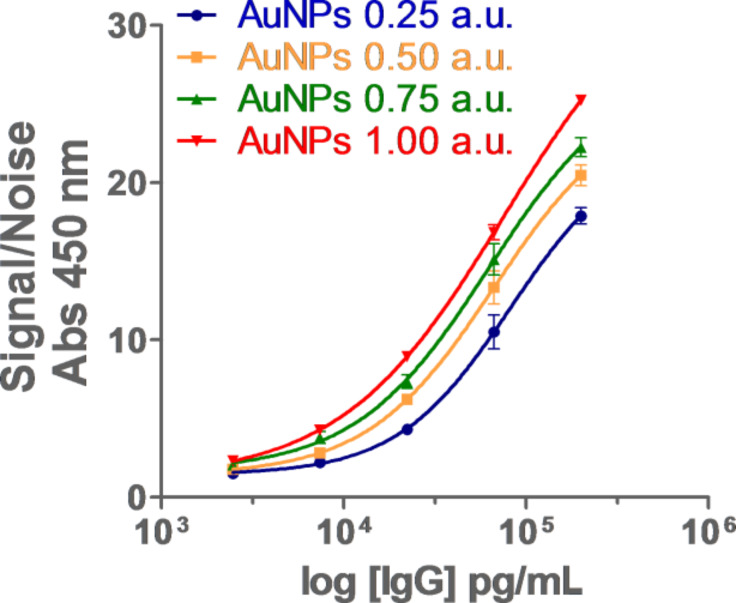
Optimization of AuNP probe concentration to be used in ELISA. Assayed concentrations: 0.25, 0.5, 0.75 and 1.00 Au. Results are normalized as S/N ratio which represents the absorbance at 450 nm of samples in the presence and absence of IgG, respectively.

Once the best functionalization strategy (adsorption) was defined, the influence of the concentration of the complex was probed in an ELISA model. As described above (Figure 2), the higher AuNP concentration, the higher the signal. Accordingly, this hypothesis was checked with four different concentrations of AuNP conjugates: 0.25, 0.5, 0.75 and 1.00 a.u. (Figure 4).

In fact, it was confirmed that the higher concentration of AuNPs, the higher the S/N. However, it was expected that doubling the concentrations of the conjugates (i.e., from 0.5 a.u. to 1 AU) would result in an increase of S/N. This effect was not observed, where the concentrations of 0.75 a.u. and 1 a.u. produced only an increase of 9% and 23% S/N in ratio at 200 ppb of IgG. Additionally, the concentration of 1 a.u. induced a higher unspecific signal. The best balance between high sensitivity and reduced utilization of AuNP probes, as well as low unspecific signal, was found at a concentration of 0.5 a.u. Moreover, this result is consistent with the literature, as published by Ambrosia and co-workers in studying the effect of AuNP complexes in enhanced ELISA for the detection of breast cancer biomarkers [12]. In this work, the authors assessed three different concentrations of AuNP probes (ranging approximately from 1.4 to 0.014 AU) discarding the highest and lowest concentrations due to unspecific signal and low signal enhancement, respectively.

### Enhanced gliadin ELISA

Once defined as the best strategy for ELISA enhancement of IgG/anti-IgG for conjugation of AuNPs to Ab, the adsorption method was tested for the detection of a real analyte, namely, gliadin. Gliadin (which can be also subdivided into α-gliadin, γ-gliadin and ω-gliadin) is a prolamin protein present in wheat gluten and one of the presumed causes of celiac disease [24]. The official detection method by Codex Alimenatarius Commission is a sandwich ELISA assay. For this reason, it was selected as a proof-of-concept for improving the detection limit based on AuNPs conjugates and application to commercial rabbit polyclonal antibody (anti-gliadin).

**Figure 5:**
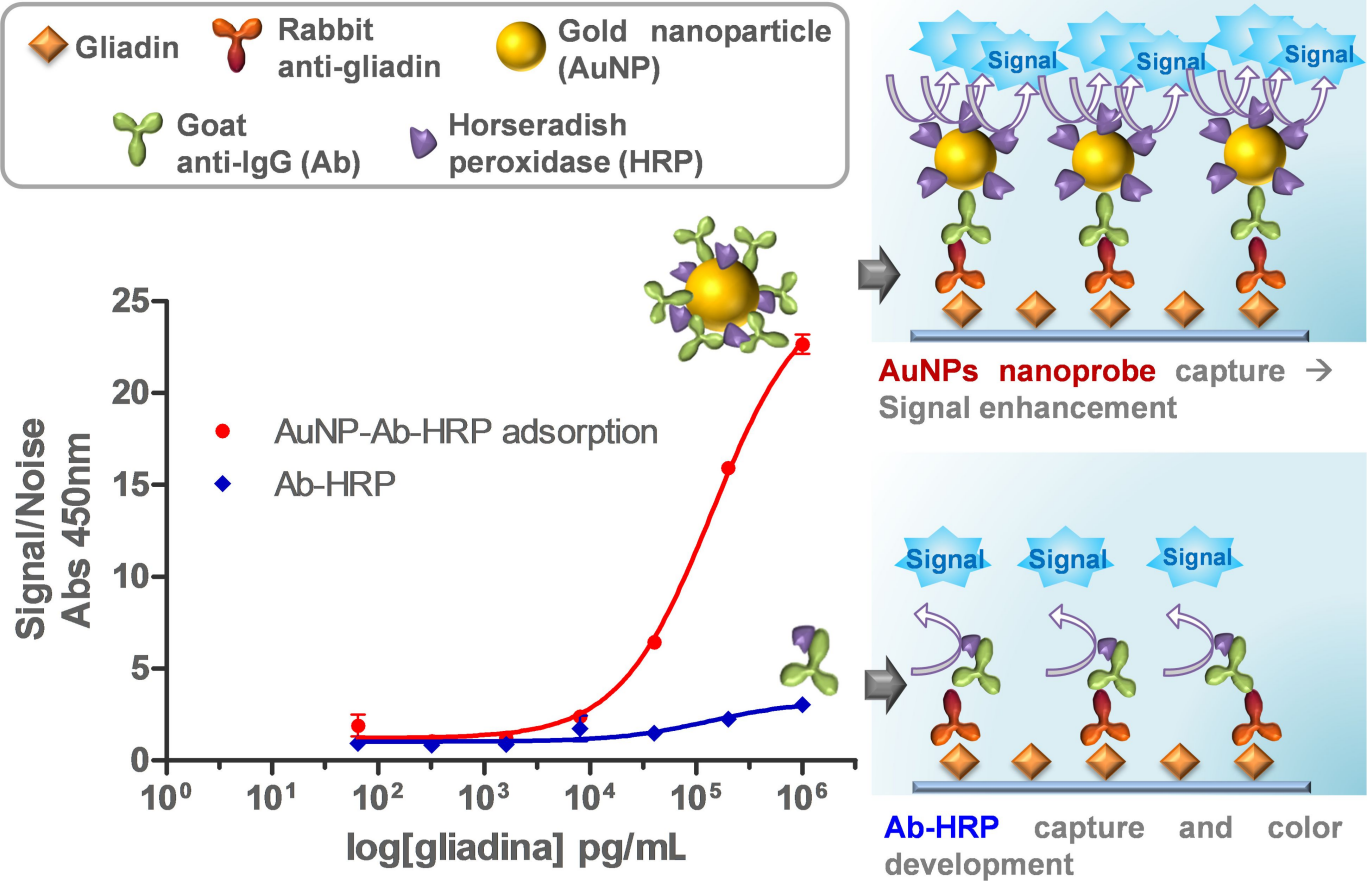
Schematic representation of gliadin detection by indirect ELISA and the basis of enhancement by means of AuNP probes. (Inset) Result of gliadin ELISA comparing AuNP probes prepared by adsorption (red curve) and Ab-HRP (blue). Results are represented as S/N ratio which represents the absorbance at 450 nm of samples in the presence and absence of IgG respectively. Coefficient of determination *R*^2^ = 0.9968 for AuNP probes and *R*^2^ = 0.7971 for Ab-HRP. Note that assayed concentrations are around the theoretical LOD of Ab-HRP.

An indirect ELISA was selected for the analysis (reference), where gliadin was coated on the ELISA plate at different concentrations (0–1 µg/mL dilutions 1:5) (Figure 5). After blocking, the primary antibody for gliadin was added at the supplier’s recommended dilution (1:5,000). Subsequently, the secondary antibody (Ab-HRP or AuNPs probes) was added at optimal dilution (i.e., 1:10,000 for commercial antibody and 0.5 a.u. for AuNPs conjugates) and recorded signals were compared. As seen in Figure 5, enhanced ELISA provides a higher signal, therefore improving the sensitivity, and also the detection limit.

The enhanced procedure resulted in more than seven times higher S/N values at 1 × 10^6^ pg/mL than regular ELISA. The LOD, estimated as the blank signal plus three times the blank standard deviation, reveals a theoretical LOD near 180 pg/mL for this enhanced ELISA, whereas conventional ELISA presents a theoretical LOD close to 500 pg/mL. The improvement of three times the LOD is similar to other works using the same functionalization strategy (adsorption) and 20 nm AuNPs [8,12]. Moreover, it should be pointed out that only a 5 min incubation with 3,3’,5,5’-tetramethylbenzidine (TMB) is needed to reach a measurable and even saturated signal (depending on target concentration), while classical ELISA often requires at least 30 min to develop the color. Therefore, this enhanced strategy could help not only for improving the sensitivity and detection limit of ELISA performance, but also for decreasing the ELISA assay time as other authors have proposed [35]. This extended assay time is recognized as one of the major handicaps nowadays of the ELISA assay [3]. In addition, this improved methodology has the potential for improving the detection of other target antigens by indirect ELISA, as AuNPs are functionalized with a universal goat secondary antibody. Another possibility is to use this method in direct ELISA by conjugating primary antibodies and HRP on AuNPs. Many of the allergen determinations by ELISA use this strategy, but more research on this is necessary to confirm this.

## Conclusion

In summary, our main objective at the start of the work was to elucidate whether a covalent loading or directional binding of biomolecules on AuNPs could lead to better results than simple direct adsorption for an enhanced ELISA application. For this purpose, four different functionalization methods of AuNPs with HRP and goat anti-rabbit IgG were used in order to enhance the ELISA sensitivity with respect to regular ELISA. The synthesized AuNPs probes were assessed in model rabbit IgG and anti-rabbit IgG ELISA by comparing the colorimetric S/N ratio. The direct adsorption method prevails as the better option with respect to the other methodologies due its performance, presenting in addition an easier preparation (no chemical steps are needed). This method was applied for improving gliadin detection by indirect ELISA. The application of AuNP probes reduced the theoretical LOD to 180 pg/mL, which is three times lower than regular ELISA, and led to an increase of at least seven times in sensitivity at level of 1 × 10^6^ pg/mL. This strategy could help to shorten ELISA assay times, making it less time consuming as well as increasing sensitivity and the LOD of the experiment. In addition, this methodology could be extended to other ELISA systems where a secondary labelled antibody is needed. Moreover, it could be a suitable methodology for combining primary antibodies with HRP, avoiding tedious chemical labelling procedures.

## Experimental

### 20 nm gold nanoparticle synthesis

All glassware was cleaned with aqua regia (HNO_3_/HCl, 3:1), rinsed with deionized water and let dry before use. 20 µL of 30% HAuCl_4_·3H_2_O was added to 95 mL of deionized water in a 100 mL flask and heated to boiling under vigorous stirring. 5 mL of 1% aqueous sodium citrate was added to the solution, changing color from yellow to dark red. The nanoparticles were maintained at boiling for 15 min after the complete color change and then removed from heat. Stirring was maintained until the flask reached room temperature. AuNPs and conjugates were characterized by dynamic light scattering (DLS) using a zeta potential analysis system (Zetasizer Nano Z, Malvern Instrumentd, Worcestershire, UK), field emission scanning electron microscope (Carl Zeiss) and UV–vis spectrophotometer (Supporting Information File 1, Figure S1).

### Gold nanoparticle functionalization

Four different strategies were assayed in this work and are schematized in Figure 1. For the direct adsorption functionalization, 133 μL of 15 mM borate buffer pH 8.7 were added to 1 mL of AuNPs synthetized as described above to adjust the pH. The appropriated amount of goat anti-rabbit IgG and horseradish peroxidase were added and allowed to react under agitation in a carrousel for 30 min. Afterwards, sucrose was incorporated to a final concentration of 5% and incubated for 30 min. Finally, 160 μL of 3% bovine serum albumin (BSA) were added and shaken for 10 min. Thereafter the sample was centrifuged (7,500*g* 30 min) to remove unbound protein and AuNPs were re-suspended in 1 mL of 2 mM borate buffer pH 8.7 containing 5% sucrose, 2% glycerol, 0.5% BSA, and 0.01% Tween. The washing step was repeated once and the AuNP probe was re-suspended in 100 μL of the mentioned borate buffer. The complex concentration was measured by absorption at 520 nm and kept at 4 °C until use.

For the directional functionalization, the protocol of Kumar and co-workers was followed with slight modifications [20]. Anti-rabbit goat IgG (Ab) and HRP were oxidized with periodate and incubated with the linker hydrazine dithiol. Briefly, 100 μL of Ab 1 mg/mL was incubated with 30 μL of 100 mM phosphate pH 7.4 and 10 μL of periodate 100 mM protected from light for 30 min. In the case of peroxidase, 200 μL of HRP 3 mg/mL were incubated with 20 μL of periodate 100 mM protected from light for 20 min. After these incubation times, 500 μL of PBS were added respectively to quench the reaction. Thereafter, 1.97 μL of 23.5 mM linker hydrazine dithiol were added and mixed for 2 h at room temperature protected from light. The proteins were buffer exchanged against phosphate buffer 10 mM pH 7.4 using a Hi-Trap desalting column using an Äkta Prime apparatus (GE-Healthcare, Upsala, Sweden). The Ab-linker and HRP-linker concentrations were measured by absorption at 280 nm and 403 nm, respectively, as well as by Bradford assay (data not shown). Afterwards, the appropriated amount of Ab-linker and HRP-linker were mixed with 1 mL of AuNPs and incubated for 20 min. Thereafter, 100 μg of m-PEG thiol were added and mixed again for 20 min. Subsequently, 100 µL of 1 mg/mL of BSA were incubated for 10 min more. Samples were centrifuged (5,000*g* 30 min) and re-suspended in 1 mL phosphate buffer 10 mM pH 7.4 containing 0.5% BSA and 0.01% Tween 20. This step was repeated twice but after the last wash, the complex was re-suspended in 400 µL. The complex concentration was measured by absorption at 520 nm and kept at 4 °C until use.

In case of the directional/adsorption functionalization, the protocol of both methods was followed with some modifications. Briefly, to 1 mL of synthesized AuNPs, 133 µL of 15 mM borate buffer pH 8.7 were added to adjust the pH. Then, the appropriate amount of Ab-linker was added to reach a final concentration of 2.25 ppm and the solution was mixed for 20 min at room temperature. After antibody incubation, the sample was mixed with nonmodified HRP to a final concentration of 144 ppm and shook for 20 min. Thereafter, sucrose (5%), BSA (0.5%), and Tween 10 (0.01%) were added to assure complex stability. The mixture was allowed to react for 10 min and purified by centrifugation at 7,500*g* for 30 min. The AuNP probe was re-suspended in 1 mL of borate buffer pH 8.7 containing 5% sucrose, 2% glycerol, and 0.01% Tween. The washing step was repeated once and the complex was re-suspended in 100 μL of mentioned buffer. The complex concentration was measured by absorption at 520 nm and kept at 4 °C until use.

Covalent functionalization was achieved using hetero-bifunctional linkers of polyethyleneglycol (PEG). In this case, AuNPs were incubated overnight with methyl-PEG-thiol (mPEG thiol, *n* = 6) and PEG-thiol acid (*n* = 7) in order to create a mixed monolayer of linker on the nanoparticle. 1 mL containing 0.075 M of mPEG thiol and 0.025 M of PEG-thiol acid was added to 100 mL of synthetized AuNPs and maintained overnight under stirring. Subsequently, the AuNPs were washed by centrifugation at 18,000*g* for 30 min and the obtained pellet was re-suspended in a smaller volume of water to arrive at a concentration factor of approximately ×30. The conjugation to antibody and peroxidase was achieved by applying the carbodiimide method to carboxylic groups of PEG-thiol acid [30]. Accordingly, 750 µL of AuNPs-PEG where added to 750 µL of a mixture of EDC/NHS 40/20 mM and incubated for 30 min at room temperature. Thereafter, AuNPs were centrifuged at 18,000*g* for 30 min and re-suspended in 1,500 µL of a solution containing 25 ppm Ab and 440 ppm HRP in borate buffer pH 8.7 and incubated for 4 h at room temperature. Finally, AuNP-Ab-HRP complexes were washed twice at 18,000*g* for 30 min and re-suspended in 300 µL Tris-HCl 20 mM pH 8.8 20% glycerol and 1% BSA. The complex concentration was measured by absorption at 520 nm and kept at 4 °C until use.

In all cases, the incubation of the proteins with AuNPs was made at room temperature stirring the mixture in a carrousel.

### Design of experiments

To build the design of experiments (DOE) matrix, some conjugations of AuNPs with antibody and HRP and simple ELISA assays were developed. For this, samples were functionalized at different ratios of HRP/Ab (1:5, 1:40, 1:75) according to the adsorption and directional procedure described above. 96-Multiwell plates were coated with a fixed concentration of rabbit IgG (1 μg/mL) 1 h at 37 °C in 10 mM carbonate buffer pH 9.6. Afterwards, these plates were washed one time with phosphate buffer saline (PBST, 0.5% Tween 20), blocked with 1% BSA in PBST and incubated with the different samples of AuNP probes for 30 min. For each HRP/Ab ratio, three different concentrations of AuNP probes (measured as the absorbance at 520 nm) were assayed, 0.05, 0.25 and 0.50 absorbance units (AU). Subsequently, the plates were washed three times with PBST and 100 μL of HRP substrate were added (TMB 0.1 mg/mL, 0.006% H_2_O_2_ in 40 mM pH 5.5 citrate buffer). After 15 min at room temperature, the reaction was stopped by adding 50 µL of 4 N H_2_SO_4_ and the absorbance was measured at 450 nm in a Synergy Mx microplate reader from Biotek.

The results were used to build a surface-of-response graphic and to determine the best HRP/Ab ratio and probe concentration in order to optimize the ELISA using DOE pro XL 2010 software from Microsoft.

### ELISA rabbit IgG probed by goat anti-IgG-HRP and AuNP conjugates

The ELISA plate was coated using different rabbit IgG concentrations (ranging 0–1 µg/mL) in carbonate buffer 10 mM pH 9.6 for 4 h at RT or overnight at 4 °C. Then the plates were washed three times with PBST and blocked using BSA 1% in PBST at 37 °C for 30 min. The plates were washed three times with PBST and incubated with 100 µL of goat anti-rabbit IgG-HRP conjugated (Ab-HRP, dilution 1:10,000) or AuNP probes (AuNP-Ab-HRP) at the appropriate concentration at 37 °C for 30 min in buffer NaPi 10 mM pH 7.4, 0.5% BSA and 0.05% Tween 20. The plates were washed four times with PBST and incubated 5 min with HRP substrate. The reaction was stopped by adding 50 µL of H_2_SO_4_ 4 N and the absorbance was measured at 450 nm in a microplate reader. For each step a volume of 100 µL was used, except for the washing step where 300 µL were used. A curve log(agonist)–response was adjusted to obtained data *y* = min + (max − min)/(1 + 10^log(EC50 − X)^).

### ELISA gliadin probed by goat anti-IgG and AuNP conjugates

The ELISA plate was coated using different gliadin concentrations ranging from 0–1 µg/mL dilutions 1:5 in carbonate buffer 10 mM pH 9.6 4 h at RT. Then, the plates were washed three times with PBST and blocked with BSA 1% in PBST at 37 °C for 30 min. The plates were washed three times with PBST and incubated with rabbit anti-gliadin antibody diluted 1:5,000 times in PBST for 30 min at room temperature. The plate was washed three times with PBST and incubated with the appropriated amount of anti-IgG-HRP (from now Ab-HRP, dilution 1:10,000) or Au probes at 37 °C for 30 min in the buffer NaPi 10 mM pH 7.4, 0.5% BSA and 0.05% Tween 20. The plates were then washed four times with PBST and incubated for 5 min with HRP substrate. The reaction was stopped by adding 50 µL H_2_SO_4_ 4 N and read at 450 nm. For each step, a volume of 100 µL was used, except for the washing step where 300 µL were used. A curve log(agonist)–response is adjusted to obtained data *y* = min + (max − min)/(1 + 10^log(EC50 − X)^).

### Materials

The BSA fraction VI for blocking was purchased from Merck. ELISA Maxisorb plates were acquired from Nunc. The linker PEG6-hydrazide aromatic dialkanedithiol was used for derivation of antibody and HRP in the directional conjugation and was obtained from NanoScience Instruments. The linkers mPEG-thiol (*n* = 6) and PEG-thiol acid (*n* = 7) for the covalent functionalization were acquired from Polypure. Rabbit IgG, polyclonal goat anti-rabbit IgG, goat anti-rabbit IgG HRP conjugated, HRP type VI, gliadin from wheat gluten, rabbit anti-gliadin and all other chemicals used were purchased from Sigma-Aldrich.

## Supporting Information

File 1 Additional figure.
