# Supplementary material for: Comparison of four functionalization methods of gold nanoparticles for enhancing the enzyme-linked immunosorbent assay (ELISA)
Source: Beilstein J Nanotechnol. 2017 Jan 25;8:244–53. doi: 10.3762/bjnano.8.27 (PMC5301989; doi:10.3762/bjnano.8.27)
Supplement: File 1 — Additional figure. [file Beilstein_J_Nanotechnol-08-244-s001.pdf]

Supporting Information

for

Comparison of four functionalization methods of gold nanoparticles for enhancing the enzyme-linked immunosorbent assay (ELISA)

Paula Ciaurriz[1], Fátima Fernández[1], Edurne Tellechea[1], Jose F. Moran[2] and Aaron C. Asensio*[1]

Address: [1]Cemitec (Multidisciplinary Center of Technologies for Industry), Polígono Mocholí, Plaza Cein 3, Noain 31110, Spain and [2]IdAB-CSIC-UPNA-GN (Institute of Agro-Biotechnology), Public University of Navarre, Campus Arrosadía s/n, Pamplona 31006, Spain

Email: Aaron C. Asensio - acabrera@cemitec.com

*Corresponding author

Additional Figure

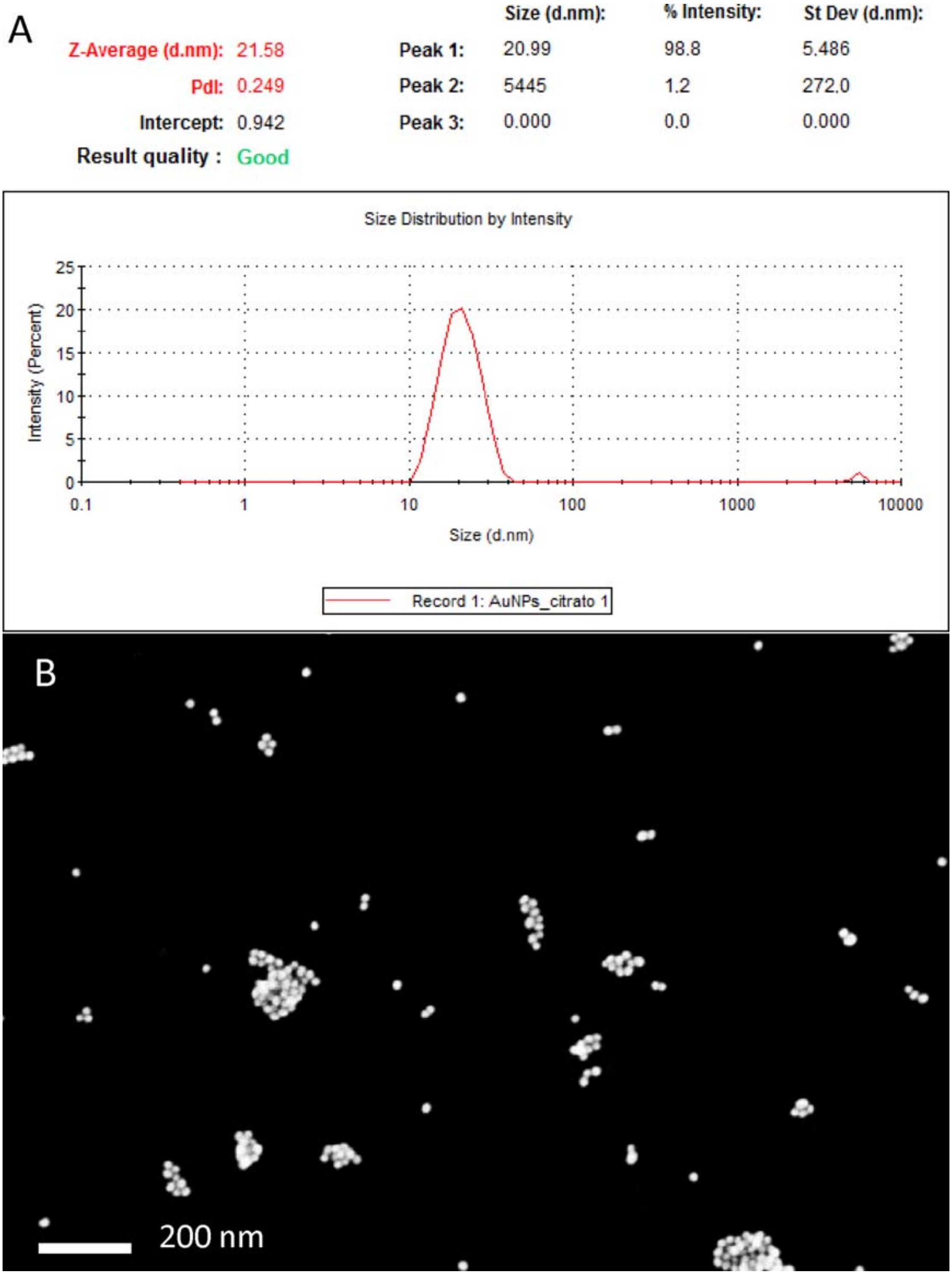

Figure S1: Characterization of nanoparticle synthesis by using DLS (A) and SEM (B). The resulting AuNPs have a spherical shape and a diameter of approximately 20 nm.
